# Associations of body size with all-cause and cause-specific mortality in healthy older adults

**DOI:** 10.1038/s41598-023-29586-w

**Published:** 2023-03-07

**Authors:** Prudence R. Carr, Katherine L. Webb, Johannes T. Neumann, Le T. P. Thao, Lawrence J. Beilin, Michael E. Ernst, Bernadette Fitzgibbon, Danijela Gasevic, Mark R. Nelson, Anne B. Newman, Suzanne G. Orchard, Alice Owen, Christopher M. Reid, Nigel P. Stocks, Andrew M. Tonkin, Robyn L. Woods, John J. McNeil

**Affiliations:** 1 School of Public Health and Preventive Medicine, Monash University, 553 St Kilda Road, Melbourne, VIC 3004 Australia; 2 Department of Cardiology, University Heart & Vascular Centre, Hamburg, Hamburg Germany; 3 German Centre for Cardiovascular Research (DZHK), Partner Site Hamburg/Kiel/Lübeck, Hamburg, Germany; 4 School of Medicine, Royal Perth Hospital, University of Western Australia, Perth, Australia; 5 Department of Pharmacy Practice and Science, College of Pharmacy; and, Department of Family Medicine, Carver College of Medicine, The University of Iowa, Iowa City, IA USA; 6 Usher Institute, The University of Edinburgh, Edinburgh, UK; 7 Menzies Institute for Medical Research, University of Tasmania, Hobart, Australia; 8 Centre for Aging and Population Health, Department of Epidemiology, University of Pittsburgh, Pittsburgh, USA; 9 School of Population Health, Curtin University, Perth, WA Australia; 10 Discipline of General Practice, Faculty of Health Sciences, University of Adelaide, Adelaide, Australia

**Keywords:** Disease prevention, Risk factors

## Abstract

In the general population, body mass index (BMI) and waist circumference are recognized risk factors for several chronic diseases and all-cause mortality. However, whether these associations are the same for older adults is less clear. The association of baseline BMI and waist circumference with all-cause and cause-specific mortality was investigated in 18,209 Australian and US participants (mean age: 75.1 ± 4.5 years) from the ASPirin in Reducing Events in the Elderly (ASPREE) study, followed up for a median of 6.9 years (IQR: 5.7, 8.0). There were substantially different relationships observed in men and women. In men, the lowest risk of all-cause and cardiovascular mortality was observed with a BMI in the range 25.0–29.9 kg/m^2^ [HR_25-29.9 vs 21–24.9 kg/m_^2^: 0.85; 95% CI, 0.73–1.00] while the highest risk was in those who were underweight [HR_BMI <21 kg/m2 vs BMI 21–24.9 kg/m2_: 1.82; 95% CI 1.30–2.55], leading to a clear U-shaped relationship. In women, all-cause mortality was highest in those with the lowest BMI leading to a J-shaped relationship (HR_BMI <21 kg/m2 vs BMI 21–24.9 kg/m2_: 1.64; 95% CI 1.26–2.14). Waist circumference showed a weaker relationship with all-cause mortality in both men and women. There was little evidence of a relationship between either index of body size and subsequent cancer mortality in men or women, while non-cardiovascular non-cancer mortality was higher in underweight participants. For older men, being overweight was found to be associated with a lower risk of all-cause mortality, while among both men and women, a BMI in the underweight category was associated with a higher risk. Waist circumference alone had little association with all-cause or cause-specific mortality risk.

*Trial registration* ASPREE https://ClinicalTrials.gov number NCT01038583.

## Introduction

A number of previous reviews on the relationship between BMI and all-cause mortality in older adults (≥ 65 years) have suggested that individuals with a BMI in the overweight range (BMI 25–29.9 kg/m^2^) (and sometimes, in those with obesity–BMI ≥ 30 kg/m^2^) had a similar or lower risk of all-cause mortality than those in the normal weight range^1,2^. Similarly, several reviews have shown that mortality tended to increase in adults at the lower end of the recommended BMI range (< 21 kg/m^2^), even after comprehensive adjustment for relevant confounders^1–5^.

These reports have predominately focused on BMI and its association with all-cause mortality, whilst much less information exists in relation to sex-specific or cause-specific mortality^1^. Moreover, in older age a reduction in lean body mass and an increase in fat mass leads to alterations in body composition that are not captured by BMI possibly making it less suited for accurately reflecting health risks associated with adiposity^6,7^. It has been suggested that waist circumference may be a better measure of adiposity and has been shown to have strong associations with mortality in younger populations^8^.

During the ASPREE clinical trial, standardised measures of weight, height and abdominal circumference were made on over 19,000 generally healthy men and women mostly aged 70 years or older. Over the subsequent 6.9 years, clinical records were reviewed (in most cases) to systematically identify the underlying cause of death. This information has provided the most comprehensive and reliable data on the relationship between different measures of obesity and cause-specific mortality yet available in older adults.

Given the increasing proportion of the population aged 60 years and above, which is projected to increase from 1 billion in 2020 to 2.1 billion by 2050^9^, it is important to understand the health risk imposed by body size and the optimal measures that can be employed to assess the risk. Using data from the ASPREE clinical trial, we therefore, explored the association of BMI and waist circumference with all-cause and cause-specific mortality to determine their relative importance as predictors of mortality from different causes in later life.

## Methods

### Study population and trial design

The ASPirin in Reducing Events in the Elderly (ASPREE) study was a large, randomized, double-blind, placebo-controlled trial investigating the effect of 100 mg aspirin on disability-free survival in apparently healthy men and women who were 70 years of age or older (or ≥ 65 years of age for African Americans and Hispanics in the US). All participants were required to be in good health, with no prior cardiovascular disease (CVD) events, dementia or major physical disability and expected to survive for at least five years. Details of the ASPREE trial and the primary results of the study have been published previously^10–13^. Briefly, from March 2010 to December 2014, 19,114 community-dwelling individuals across Australia (n = 16,703) and the US (n = 2411), gave written informed consent and were randomized. In 2017, ASPREE transitioned to a longitudinal, observational follow up study of the trial participants who at the time of this report had been followed for a median of 6.9 years since study entry (IQR: 5.7, 8.0).

The trial was conducted according to the Australian National Statement on Ethical Conduct in Human Research, the Australian Code for the Responsible Conduct of Research, the 2008 Declaration of Helsinki, and the International Conference on Harmonization Good Clinical Practice E6 and was approved by institutional review boards at all sites. All participants provided written informed consent. The ASPREE study is registered on ClinicalTrials.gov (Trial registration number: ASPREE ClinicalTrials.gov number NCT01038583, 24/12/2009) and the International Standard Randomised Controlled Trial Number Registry (ISRCTN83772183). This study was reviewed and approved by the Monash University Human Research Ethics Committee (Project No. 24743).

### Study measurements

All ASPREE participants completed two baseline visits to finalize their eligibility for the study, and after randomization were assessed annually by trained study staff. At baseline and every annual study visit, participants underwent comprehensive evaluations of physical measures, collection of anthropometric and laboratory measurements, medical morbidities, lifestyle and socio-demographic factors, concomitant prescription medications, and other related health parameters. Full details on the ascertainment of these study measurements have been described in detail previously^14,15^.

### Exposure assessments

At study enrolment, anthropometric measures which included body weight, height and waist circumference, were taken by trained study staff according to a strict protocol. Body weight was measured in kilograms, with the participant in bare feet, wearing light clothing only. Standing height was measured in meters, with the participant in bare feet, looking straight ahead, with as many body points against the wall as possible, using a wall-mounted stadiometer, where available, but if not, using a right-angled ruler to record the height before measurement with a tape measure. BMI was calculated as weight divided by height squared (kg/m^2^).

Measurement of waist circumference with a ‘Figure Finder Tape Measure’ was made on bare skin, with the participant standing. The measurement was taken mid-way between the unclothed crest of the hip and the lowest rib, keeping the tape measure horizontal, ensuring the participant was breathing normally, with arms resting at their sides. All study sites were monitored regularly to ensure staff competency and adherence to standardized operating procedures.

### Ascertainment of outcomes

Full details on the ascertainment of death have been described in detail previously^10^. Briefly, in most cases, deaths were identified during the course of routine trial activity, by review of health records, or by the next of kin or close contact who notified the trial centre. In all cases, notifications of death required confirmation from two independent sources (the family, the primary care physician, or a public death notice). At the end of the trial, participants who had withdrawn or were lost to follow-up, were linked to the National Death Index in the relevant country. After a notification of death was confirmed, deaths were classified according to the underlying cause by adjudicators who were unaware of trial-group assignments. In the current analyses, deaths were classified as death from any cause and death related to specific causes including deaths from ischaemic CVD (any ischemic event [myocardial infarction, other coronary heart disease, sudden cardiac death, or ischemic stroke]), cancer (deaths that were related to primary or metastatic cancer), or other causes (deaths that were related to causes that were non-cancer or non-CVD [e.g. sepsis, chronic lung disease, dementia]).

### Statistical analyses

Cox proportional hazards regression was used to calculate sex-specific hazard ratios (HRs) and 95% confidence intervals (CIs) to assess the relationship between the baseline anthropometric measures and all-cause and cause-specific mortality. For cause-specific mortality, participants were censored for other causes at time of death to obtain cause-specific hazard ratios. In the current analyses, BMI was used as a categorical variable to enhance comparability with existing studies, using specific cut points for older adults based on a recent meta-analysis which summarized the evidence on the association between BMI and all-cause mortality in adults ≥ 65 years^1^. These cut offs were: BMI < 21.0, 21.0–24.9 (reference group), 25.0–29.9, 30.0–34.9, and ≥ 35.0 kg/m^2^. Waist circumference was categorized into sex-specific quintiles (Q1 to Q5), with the second quintile serving as the reference group, to capture the impact at the extremes and because the second quintile contained (approximately) the WHO recommended cut-offs^17^. Each analysis was adjusted for age (Model 1), then additionally for potential confounders selected a priori including education level (< 12 years, ≥ 12 years), smoking status (current, former/never), diabetes status (yes, no), study treatment arm (aspirin, placebo), living situation (at home alone, with others), alcohol consumption (current, former/never), and the longest amount of time walking outside the home without sitting down to rest (less than 10 min, 10–15 min, 16–30 min, > 30 min), as a proxy for physical activity (Model 2). We also used restricted cubic splines with three knots at the 10^th^, 50th, and 90th percentiles to model the association of continuous BMI and waist circumference with mortality separately for men and women. Figures are presented for age-adjusted all-cause mortality and cause-specific mortality.

For further comparison with other studies, the WHO BMI recommended cut-offs were also used, with underweight defined as BMI < 18.5 kg/m^2^, normal weight as BMI 18.5–24.9 kg/m^2^ (reference group), overweight as BMI 25.0–29.9 kg/m^2^ and general obesity as BMI ≥ 30 kg/m^2^^16^. Waist circumference was also assessed as a dichotomized variable using the WHO recommended cut offs for high disease risk (< 88 cm or ≥ 88 cm in women; < 102 cm or ≥ 102 cm in men) for further comparison with previous studies^17^. We also explored the interaction between the BMI categories and age. In sensitivity analyses, we repeated the main analyses after excluding those participants with a history of diabetes and after excluding the US and Australian minorities because the BMI mortality association may differ according to race or ethnicity^18^.

All statistical tests were two sided, and we considered *P* < 0.05 to determine statistical significance. Confidence intervals and P values were not adjusted for multiple comparisons. Statistical analyses were performed using R version 4.0.2 (R Core Team, 2020).

## Results

### Study participants

**Figure 1:**
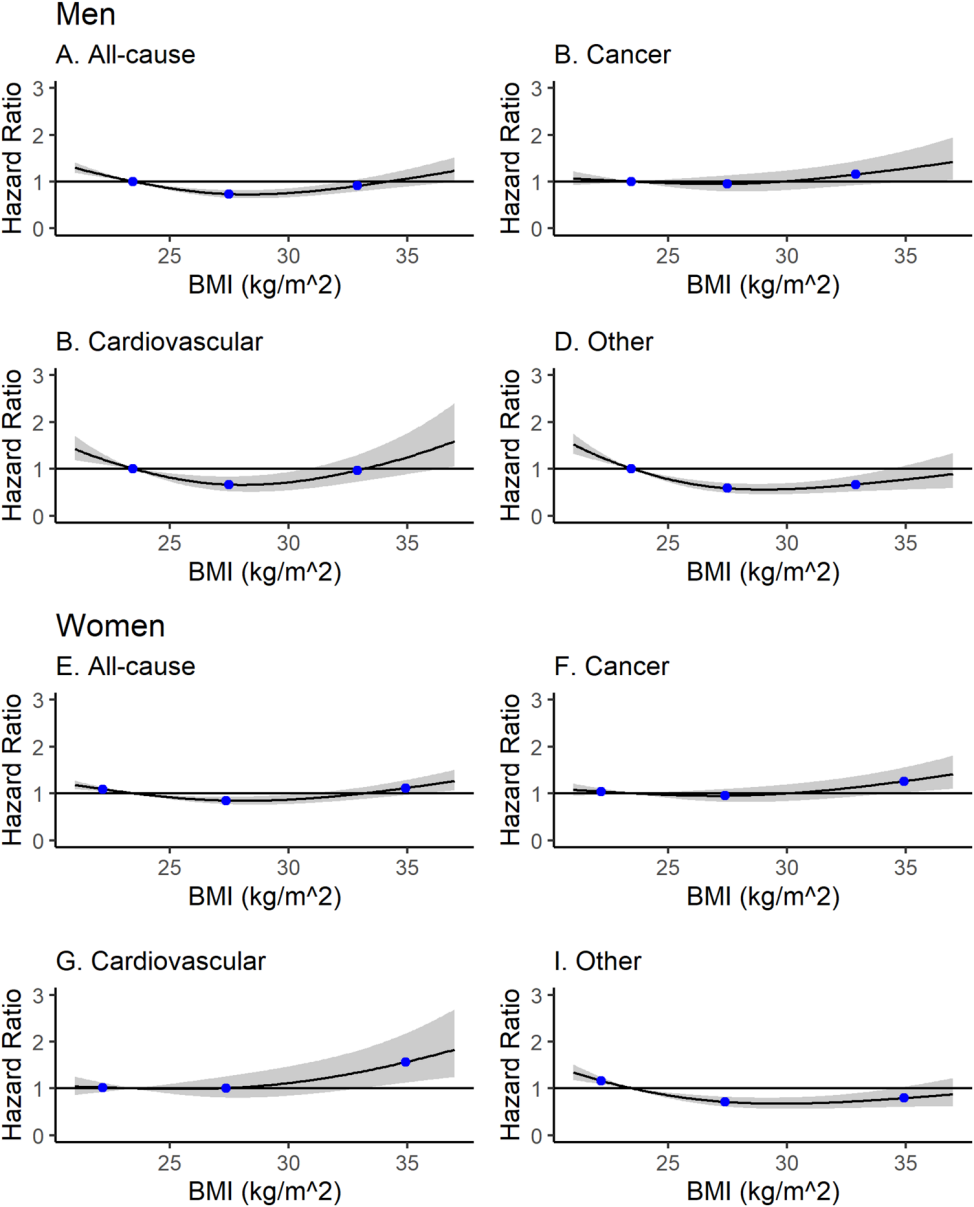
Hazard ratio for all-cause mortality, cancer mortality, cardiovascular mortality, and other mortality as a function of body mass index (BMI) in men (**A**–**D**) and women (**E**–**H**). Restricted cubic spline with knots at 23.5, 27.5, 32.9 (men) and 22.2, 27.4, 34.9 (women). Hazard ratios are indicated by the solid lines and 95% confidence intervals by shaded areas.

After participants with missing covariate information or those missing both body size measures were excluded, we analysed data from 18,209 participants (95.3% of the ASPREE study population) (Appendices Fig. 1). Among them, 56% were women, 10% had a history of diabetes, and 31% used HMG CoA reductase inhibitors (“statins”) at baseline. The mean age was 75.1 (SD, 4.5) years, and the mean BMI was 27.9 (SD, 3.9) kg/m^2^ in men and 28.1 (SD, 5.2) kg/m^2^ in women. Mean waist circumference was 101.9 (SD, 10.8) cm in men and 93.1 (SD, 12.9) cm in women (Table 1). During a median follow up of 6.9 years (IQR: 5.7, 8.0)., we identified 1762 deaths (men: 966; women: 796).

**Table 1:** Baseline characteristics of the ASPREE participants included in the current analyses.

	Overall	Men	Women
	N = 18,209	n = 8054	n = 10,155
Age (years, mean (SD))	75.1 (4.5)	74.9 (4.4)	75.2 (4.6)
Country (n (%))			
Australia	15,991 (87.8)	7288 (90.5)	8703 (85.7)
United States	2218 (12.2)	766 (9.5)	1452 (14.3)
Race/ethnicity (n (%))			
White/Aus	15,662 (86.0)	7106 (88.2)	8556 (84.3)
White/US	1033 (5.7)	336 (4.2)	697 (6.9)
Black	804 (4.4)	284 (3.5)	520 (5.1)
Hispanic	445 (2.4)	191 (2.4)	254 (2.5)
Other	265 (1.5)	137 (1.7)	128 (1.3)
Weight in kg (mean (SD))^1^	76.9 (14.9)	83.8 (13.2)	71.4 (13.8)
Height in m (mean (SD))^2^	1.7 (0.1)	1.7 (0.1)	1.6 (0.1)
BMI in kg/m^2^ (mean (SD))^3^	28.0 (4.7)	27.9 (3.9)	28.1 (5.2)
Waist circum. in cm (mean (SD))^4^	97.0 (12.8)	101.9 (10.8)	93.1 (12.9)
Randomized to aspirin (n (%))	9074 (49.8)	4021 (49.9)	5053 (49.8)
Baseline statin use (n (%))	5652 (31.0)	2228 (27.7)	3424 (33.7)
Current smoker (n (%))	691 (3.8)	366 (4.5)	325 (3.2)
Current alcohol drinker (n (%))	14,064 (77.2)	6732 (83.6)	7332 (72.2)
Diabetes (n (%))*	1891 (10.4)	990 (12.3)	901 (8.9)
Systolic BP (mean (SD))	139.2 (16.5)	141.1 (15.8)	137.7 (16.8)
Diastolic BP (mean (SD))	77.3 (10.0)	78.1 (9.6)	76.6 (10.2)
Education level (n (%))			
< 12 years	8174 (44.9)	3515 (43.6)	4659 (45.9)
≥ 12 years	10,035 (55.1)	4539 (56.4)	5496 (54.1)
Living situation (n (%))			
At home alone	5929 (32.6)	1654 (20.5)	4275 (42.1)
With others	12,280 (67.4)	6400 (79.5)	5880 (57.9)
Longest amount of time walking outside home without any rest (last 2 weeks)			
< 10 min	535 (2.9)	230 (2.9)	305 (3.0)
10–15 min	1800 (9.9)	711 (8.8)	1089 (10.7)
16–30 min	4140 (22.7)	1645 (20.4)	2495 (24.6)
More than 30 min	11,734 (64.4)	5468 (67.9)	6266 (61.7)

Number of participants missing baseline measurement for the following variables: ^1^n = 35, ^2^n = 28, ^3^n = 60, ^4^n = 172.

SD, standard deviation; BMI, body mass index; BP, blood pressure.

*Diabetes defined as a self-report, fasting glucose ≥ 126 mg/dL, or receiving pharmacologic treatment for diabetes (regardless of fasting glucose level).

A distribution of the baseline characteristics by BMI and waist circumference in men and women is shown in Appendices Tables 1, 2, 3, 4. In general, men and women with a higher BMI, had higher use of statins and antihypertensive medication at baseline and were more likely to have diabetes. Men and women with a higher BMI tended to consume less alcohol, and spent less time walking outside the home without any rest (Appendices Tables 1 and 2). A similar pattern was also seen for men and women with a higher waist circumference (Appendices Tables 3 and 4).

**Table 2:** Hazard ratio (95% CI) of all-cause mortality according to baseline body mass index (BMI) in men and women (n = 18,149).

BMI (kg/m^2^)	Men				Women			
	No. of deaths/No. of pts	Incidence rate per 1000py	Model 1* HR (95% CI)	Model 2^ HR (95% CI)	No. of deaths/No. of pts	Incidence rate per 1000py	Model 1* HR (95% CI)	Model 2^ HR (95% CI)
BMI < 21.0	40/153	42.68	1.97 (1.41, 2.76)	1.82 (1.30, 2.55)	76/541	20.71	1.67 (1.28, 2.18)	1.64 (1.26, 2.14)
BMI 21.0–24.9	233/1646	21.59	Ref	Ref	197/2469	11.66	Ref	Ref
BMI 25.0–29.9	458/4183	16.50	0.84 (0.71, 0.98)	0.85 (0.73, 1.00)	287/3959	10.70	0.98 (0.81, 1.17)	0.97 (0.81, 1.17)
BMI 30.0–34.9	183/1633	17.05	0.99 (0.82, 1.21)	0.95 (0.78, 1.16)	150/2153	10.32	1.05 (0.85, 1.30)	0.98 (0.79, 1.22)
BMI 35.0 +	48/417	17.81	1.16 (0.85, 1.58)	0.98 (0.71, 1.35)	84/995	12.90	1.46 (1.12, 1.88)	1.27 (0.98, 1.66)

*Model 1: Adjusted for age.

^Model 2: Adjusted for age, smoking status, aspirin treatment arm, diabetes status (yes, no), level of education (< 12 years, ≥ 12 years), living status (at home alone, with others), alcohol consumption (current alcohol consumption, former/never), and longest amount of time walking outside home without any rest.

BMI, body mass index; py, person years; Ref., reference.

**Table 3:** Hazard ratio (95% CI) of all-cause mortality according to baseline waist circumference in men and women (n = 18,037).

Waist circumference^1^	Men				Women			
	No. of deaths/No. of pts	Incidence rate per 1000py	Model 1* HR (95% CI)	Model 2^ HR (95% CI)	No. of deaths/No. of pts	Incidence rate per 1000py	Model 1* HR (95% CI)	Model 2^ HR (95% CI)
Quintile 1	225/1668	20.56	1.32 (1.09, 1.59)	1.30 (1.07, 1.58)	175/2087	12.28	1.22 (0.98, 1.53)	1.22 (0.97, 1.53)
Quintile 2	193/1828	15.82	Ref	Ref	137/2027	9.90	Ref	Ref
Quintile 3	192/1594	18.27	1.17 (0.96, 1.43)	1.15 (0.94, 1.40)	164/2127	11.38	1.16 (0.93, 1.46)	1.15 (0.92, 1.45)
Quintile 4	168/1400	18.19	1.26 (1.03, 1.55)	1.20 (0.98, 1.48)	138/1985	10.37	1.12 (0.89, 1.42)	1.07 (0.84, 1.36)
Quintile 5	176/1514	17.87	1.33 (1.08, 1.63)	1.20 (0.97, 1.47)	166/1807	13.83	1.56 (1.25, 1.96)	1.44 (1.14, 1.81)

^1^Waist circumference Quintile Range (Mean) values for Men (cm): Q1: 56–93 (88.13); Q2: 94–99 (96.67), Q3:100–104 (101.97), Q4:105–110 (107.31), Q5: 111–153 (118.15); Female: Q1: 44–82 (76.15), Q2: 83–89 (86.13), Q3: 90–96 (92.95), Q4: 97–104 (100.2), Q5: 105–180 (112.77).

*Model 1: Adjusted for age.

^Model 2: Adjusted for age, smoking status, aspirin treatment arm, diabetes status (yes, no), level of education (< 12 years, ≥ 12 years), living status (at home alone, with others), alcohol consumption (current alcohol consumption, former/never), and longest amount of time walking outside home without any rest.

py, person years; Ref., reference.

**Table 4:** Hazard ratio (95% CI) of cause-specific mortality^1^ according to baseline BMI and waist circumference in men and women (BMI: n = 18,149, WC: n = 18,037).

BMI (kg/m^2^)	Cancer death			Cardiovascular death			Other death		
	No. events/No. of pts	Incidence rate per 1000 py	HR^2^ (95% CI)	No.events/ No. of pts	Incidence rate per 1000 py	HR^2^ (95% CI)	No.events/No. of pts	Incidence rate per 1000 py	HR^2^ (95% CI)
Men									
< 21.0	10/153	10.67	1.12 (0.58, 2.16)	10/153	10.67	2.15 (1.08, 4.27)	19/153	20.27	2.34 (1.41, 3.86)
21.0–24.9	94/1646	8.71	Ref	49/1646	4.54	Ref	86/1646	7.97	Ref
25.0–29.9	228/4183	8.21	1.02 (0.80, 1.3)	84/4183	3.03	0.74 (0.52, 1.06)	138/4183	4.97	0.72 (0.55, 0.94)
30.0–34.9	93/1633	8.66	1.14 (0.85, 1.52)	39/1633	3.63	0.97 (0.63, 1.50)	51/1633	4.75	0.76 (0.54, 1.09)
≥ 35.0	25/417	9.28	1.20 (0.76, 1.90)	14/417	5.19	1.38 (0.74, 2.57)	9/417	3.34	0.53 (0.26, 1.06)
Women									
< 21.0	26/541	7.09	1.43 (0.92, 2.24)	10/541	2.73	1.15 (0.57, 2.32)	37/541	10.08	1.94 (1.31, 2.88)
21.0–24.9	80/2469	4.73	Ref	36/2469	2.13	Ref	77/2469	4.56	Ref
25.0–29.9	147/3959	5.48	1.21 (0.92, 1.60)	54/3959	2.01	1.01 (0.66, 1.54)	84/3959	3.13	0.73 (0.53, 0.99)
30.0–34.9	71/2153	4.89	1.11 (0.80, 1.53)	32/2153	2.20	1.25 (0.77, 2.03)	46/2153	3.17	0.77 (0.53, 1.11)
≥ 35.0	43/995	6.60	1.53 (1.04, 2.26)	18/995	2.76	1.65 (0.92, 2.99)	20/995	3.07	0.79 (0.47, 1.32)
Waist circumference									
Men									
Q1	87/1668	7.95	1.04 (0.78, 1.4)	48/1668	4.39	1.78 (1.13, 2.82)	86/1668	7.86	1.41 (1.03, 1.94)
Q2	93/1828	7.62	Ref	30/1828	2.46	Ref	68/1828	5.57	Ref
Q3	104/1594	9.9	1.29 (0.97, 1.71)	36/1594	3.43	1.38 (0.85, 2.24)	47/1594	4.47	0.80 (0.55, 1.16)
Q4	78/1400	8.44	1.14 (0.84, 1.54)	39/1400	4.22	1.82 (1.12, 2.93)	50/1400	5.41	1.04 (0.72, 1.50)
Q5	86/1514	8.73	1.17 (0.87, 1.58)	40/1514	4.06	1.76 (1.09, 2.86)	50/1514	5.08	1.01 (0.70, 1.47)
Women									
Q1	68/2087	4.77	0.96 (0.68, 1.34)	26/2087	1.82	0.86 (0.51, 1.46)	76/2087	5.33	1.97 (1.33, 2.94)
Q2	68/2027	4.91	Ref	29/2027	2.10	Ref	36/2027	2.60	Ref
Q3	75/2127	5.2	1.07 (0.77, 1.48)	36/2127	2.50	1.18 (0.73, 1.93)	52/2127	3.61	1.38 (0.90, 2.11)
Q4	69/1985	5.19	1.07 (0.76, 1.50)	25/1985	1.88	0.93 (0.54, 1.60)	43/1985	3.23	1.28 (0.82, 2.00)
Q5	82/1807	6.83	1.43 (1.03, 1.99)	30/1807	2.50	1.28 (0.76, 2.16)	51/1807	4.25	1.68 (1.08, 2.59)

Waist Circumference Quintile Range (Mean) values for men: Q1:56–93 (88.13); Q2:94–99 (96.67), Q3:100–104 (101.97), Q4:105–110 (107.31), Q5: 111–153 (118.15); women: Q1: 44–82 (76.15), Q2: 83–89 (86.13), Q3: 90–96 (92.95), Q4: 97–104 (100.2), Q5: 105–180 (112.77).

^1^Death related to specific causes including cancer (deaths that were related to primary or metastatic cancer), deaths from ischaemic CVD (any ischemic event [myocardial infarction, other coronary heart disease, sudden cardiac death, or ischemic stroke]), or other causes (deaths that were related to causes that were non-cancer or non-CVD [e.g. sepsis, chronic lung disease, dementia]).

^2^HRs adjusted for age, smoking status, aspirin treatment arm, diabetes status (yes, no), level of education (< 12 years, ≥ 12 years), living status (at home alone, with others), alcohol consumption (current alcohol consumption, former/never), and longest amount of time walking outside home without any rest.

BMI, body mass index; CI, confidence interval; HR, hazard ratio; py, person years; Ref., reference.

### All-cause mortality

The highest risk of all-cause mortality was seen in underweight men and women. In multivariable adjusted models, a BMI below 21 kg/m^2^ was associated with an 82% higher risk in men and a 64% higher risk in women compared with those with a BMI of 21–24.9 kg/m^2^ (HR_men_:1.82, 95% CI 1.30–2.55; HR_women_:1.64, 95% CI 1.26–2.14) (Table 2).

Men with a BMI in the range 25.0–29.9 kg/m^2^ had the lowest risk of all-cause mortality (HR_men_:0.85, 95% CI 0.73–1.00), while in women there was little difference in mortality risk amongst those with a BMI in the range 21–35 kg/m^2^ (Table 2). Restricted cubic spline analysis confirmed these results, as did an analysis using WHO recommended BMI cut-offs (Fig. 1, Appendices Table 5).

**Figure 2:**
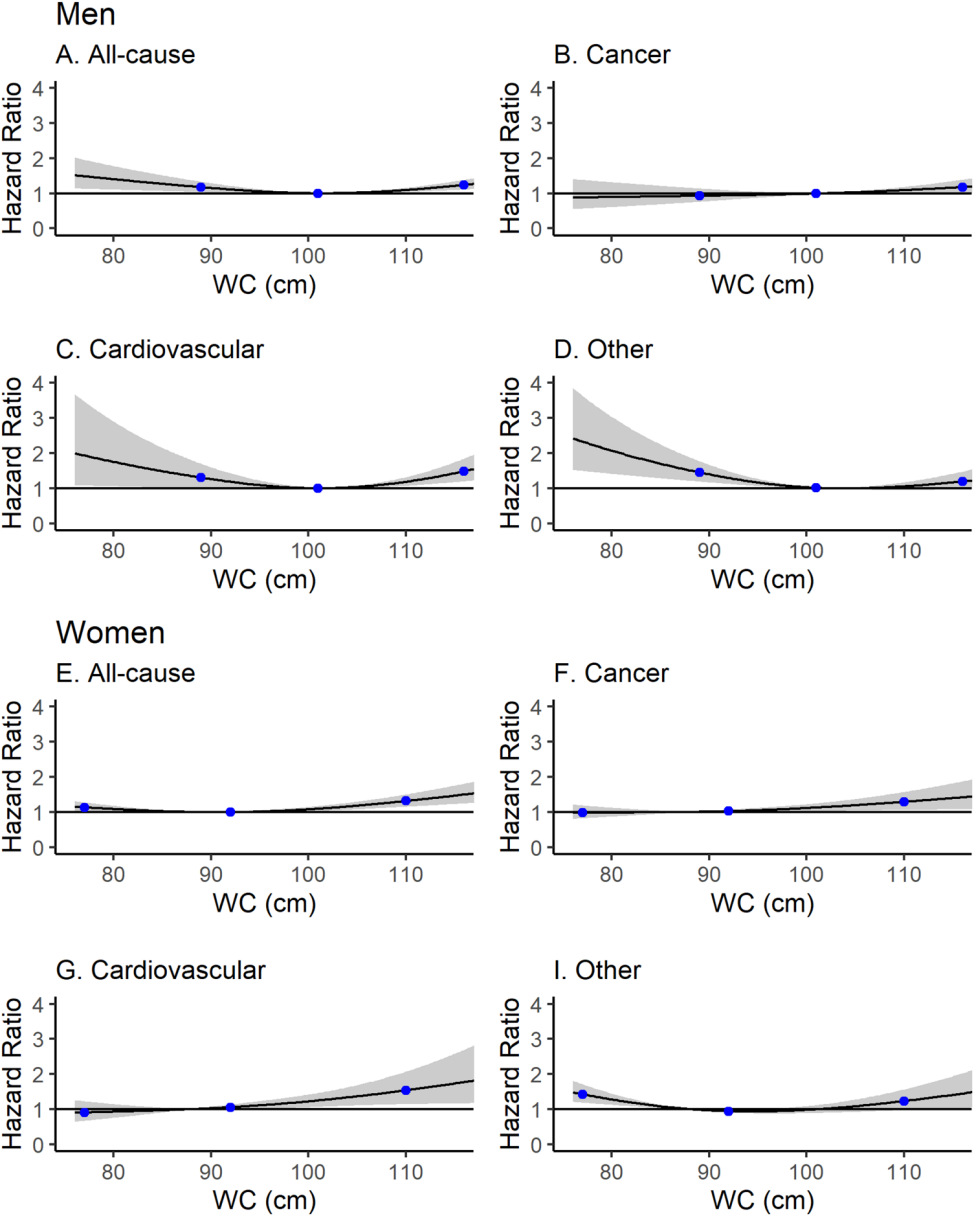
Hazard ratio for all-cause mortality, cancer mortality, cardiovascular mortality, and other mortality as a function of waist circumference (WC) in men (**A**–**D**) and women (**E**–**H**). Restricted cubic spline with knots at 89, 101, 116 cm (men) and 77, 92, 110 cm (women). Hazard ratios are indicated by the solid lines and 95% confidence intervals by shaded areas.

Waist circumference showed a weaker U-shaped relationship with all-cause mortality in both men and women with a modest increase in risk in both the lowest and highest quintile (Table 3). Restricted cubic splines and an analysis using the WHO waist circumference cut-offs again showed a similar pattern (Fig. 2, Appendices Table 6).

### Cause-specific mortality

The associations of BMI and waist circumference with cause-specific mortality (cancer death, cardiovascular death and non-cardiovascular non-cancer causes of death) are shown in Table 4 and the restricted cubic spline analyses in Figs. 1, 2.

*Cancer mortality:* No association was observed between BMI or waist circumference and cancer mortality in either men or women, apart from a small increase in risk at the upper extreme of BMI (≥ 35 kg/m^2^) and waist circumference (Quintile 5) among women (Table 4).

*Cardiovascular mortality:* In men, similar to the relationship with all-cause mortality there was a strong U-shaped relationship between BMI and waist circumference with cardiovascular death. In multivariable adjusted models, a low BMI (< 21 kg/m^2^) was associated with a higher risk of cardiovascular death in men, compared with those with a BMI of 21–24.9 kg/m^2^ (HR_cardiovascular death_:2.15, 95% CI 1.08–4.27), whilst the lowest risk was amongst those with a BMI in the range 25-30 kg/m^2^ (Fig. 1, Table 4). For waist circumference the lowest risk was found in those close to the upper cut-off of the WHO recommendations (102 cm) for men (Table 4, Fig. 2). In women, there was no evidence of a U-shaped relationship between BMI or waist circumference and cardiovascular mortality but mortality increased at the upper end of the distribution of both (Table 4, Fig. 1 and 2).

*Non-cancer, non-cardiovascular mortality:* ‘Other deaths’ demonstrated a weak U-shaped relationship, most prominently with increased risk at the lowest extremes of BMI and waist circumference (Table 4, Fig. 1 and 2).

### Sensitivity analyses

In sensitivity analyses excluding men and women with a history of diabetes (n = 1891), and excluding US and Australian minorities (n = 1514), the hazard ratios of all-cause and cause-specific mortality according to BMI and waist circumference were essentially unchanged (data not shown). We did not observe a significant interaction between BMI and age (*P* interaction > 0.05) (results not shown).

## Discussion

In this cohort of more than 18,000 relatively healthy older adults followed for almost 7 years, the principal finding was that the lowest all-cause and cardiovascular mortality occurred in men and women whose BMI and waist circumference was substantially higher than considered normal in current healthy weight guidelines. In men, the relationships were U-shaped with the highest mortality risks amongst those at the bottom and the top of the distribution. In women the increased all-cause and cardiovascular mortality risks were largely confined to the highest BMIs and waist circumferences. The U-shape relationship between mortality and BMI was stronger than the relationship with waist circumference. Amongst cause-specific mortality risks, body size parameters showed a weak relationship to cancer mortality and did not appear to explain the increased mortality amongst underweight individuals. Deaths from causes other than cancer or CVD were increased predominantly in those who were underweight.

These findings generally support the findings of a recent meta-analysis relating BMI to all-cause mortality in older populations^1^. In that review, Winter et al. described increased all-cause mortality amongst individuals aged 65 years and older whose BMI was below 23 kg/m^2^ and a minimum at a BMI of 28 kg/m^2^^1^. Currently, for adults, the WHO considers a BMI ≥ 25 kg/m^2^ as indicative of overweight and ≥ 30 kg/m^2^ as obese without reference to age^16^. A previous meta-analysis of studies of waist circumference and mortality showed a stronger relationship with all-cause, cardiovascular and cancer mortality than was identified in our study^19^. This report of 29 prospective cohort studies involving older adults aged 65–74 years noted that an increased waist circumference was associated with an increased risk of all-cause mortality, CVD mortality and cancer mortality^19^. However, another meta-analysis found no significant association in a subgroup analysis of studies with participants older than 60 years (HR, 1.03; 95% CI, 0.98–1.08, n = 14 studies)^8^. Many of the individual studies included in these meta-analyses were small, undertaken in past decades when modern preventive interventions were limited and/or used self-reported measures of body size^1,19^. Moreover, a substantial proportion were initiated at a time when CVD dominated as a cause of death in high income countries^20^. By contrast, the results from the ASPREE study involved a large contemporary population with objective anthropometric measures and causes of death principally adjudicated from medical records. The study provides the most comprehensive assessment to date of the relationship of BMI and waist circumference to all-cause and cause-specific mortality in both men and women. By studying a generally healthy population, free of past CVD or other life-threatening conditions, the relationships were unlikely to have been distorted by serious illnesses.

The principal sex differences were seen in the relationships between all-cause and cardiovascular mortality. In men, but not women, there was an increased risk of mortality amongst those at the lowest end of BMI and waist circumference. This led to a substantially greater U-shaped relationship in men than in women, particularly regarding BMI, and this relationship persisted after adjustment for multiple confounding factors. The increase in mortality from non-cancer non-cardiovascular causes amongst those with low BMI or waist circumference may reflect the impact of frailty and prefrailty in this population.

The stronger relationship between BMI and mortality compared to waist circumference and mortality was unexpected. While BMI is the most commonly used clinical and population measure of body size, it is only a rough guide to body fatness. Waist circumference better reflects visceral adiposity which has been positively and significantly associated with a higher risk of all-cause mortality in younger populations^8^. It has therefore been advocated as a more appropriate health measure^8,21^. However, given the current findings, there is strong argument that findings from middle aged persons cannot be generalised to older adults.

The relationship between body size and health is relevant to the advice given by clinicians and public health spokespeople who commonly provide a strong recommendation to overweight or obese patients to lose weight to reduce their future risk of CVD and cancer. Our findings add to the mounting evidence that the current ‘healthy weight range’ may not be suitable for older adults and strong steps to encourage weight loss in those moderately overweight or obese requires further evaluation. Nor is there a strong indication from these results for clinicians to rely on waist circumference rather than BMI when providing health advice. However, despite the findings of this study, overweight and obesity have various other negative health consequences that must also factor into the advice provided by clinicians^16,22^.

This study also has some potential limitations that require discussion. Firstly, the results of this study are most relevant to a largely Caucasian population drawn from communities with access to universal healthcare, as reflected by the high utilisation of statins and antihypertensive drugs. The high frequency of preventive medication might have blunted some adverse effects of overweight and obesity. Secondly, the population studied was drawn from volunteers for a clinical trial who are likely to have been more attentive to maintaining a healthy lifestyle that others in the community. The results may have limited relevance for South Asian, Chinese or Japanese adults where the population distributions of BMI and waist circumference may differ^18^. Thirdly, although we did conduct multiple analyses in this current study, we did not adjust for multiple comparisons and therefore cannot rule out chance findings. However, the focus of our analyses was on the overall patten of the relationship between BMI & waist circumference with mortality, rather than examining the significance at any one specific BMI or waist circumference value. Finally, due to the small group sizes, we had limited numbers of participants at the extremes of the body size measures, so some results should be interpreted with caution.

## Conclusions

In summary, this study found that in this older population, BMI has a significantly stronger relationship than waist circumference to both all-cause and cardiovascular mortality. In men, the lowest mortality was in those whose BMI was in the overweight range and the highest was amongst those who were underweight at entry to the study. As a result, there was a strong U-shaped relationship between BMI and all-cause and cardiovascular mortality in older men but less so in older women. Waist circumference showed a weaker relationship with mortality risk. Cancer mortality was unrelated to either measure of body size except at the upper extreme. This information may help inform the advice provided by primary care physicians, particularly to moderately overweight men.

## Supplementary Information

Supplementary Information.

## Data Availability

The data underlying this article can be shared on reasonable request addressed to ASPREE.AMS@monash.edu.
