# Supplementary material for: Associations of body size with all-cause and cause-specific mortality in healthy older adults
Source: Sci Rep. 2023 Mar 7;13:3799. doi: 10.1038/s41598-023-29586-w (PMC9992380; doi:10.1038/s41598-023-29586-w)
Supplement: Supplementary file 1 — Supplementary Information. [file 41598_2023_29586_MOESM1_ESM.docx]

**Associations of body size with all-cause and cause-specific mortality in healthy older adults: Supplementary Material**

Missing BMI and WC at baseline
n = 24

Missing walking time at baseline
n = 880

Missing education at baseline
n = 1

**Appendices Figure 1.** ASPREE participants included in the analyses

**Appendices Table 1.** Baseline characteristics of the ASPREE participants by BMI category in women (n = 10,117)

|  | **BMI < 21.0** | **BMI 21.0-24.9** | **BMI 25.0-29.9** | **BMI 30.0-34.9** | **BMI ≥35.0** |
| --- | --- | --- | --- | --- | --- |
|  | n=541 | n=2469 | n=3959 | n=2153 | n=995 |
| Age (mean (SD)) | 76 (5.0) | 75.7 (4.8) | 75.4 (4.6) | 74.7 (4.3) | 74.0 (4.1) |
| Country (n (%)) |  |  |  |  |  |
| Australia | 464 (85.8) | 2174 (88.1) | 3426 (86.5) | 1820 (84.5) | 783 (78.7) |
| United States | 77 (14.2) | 295 (11.9) | 533 (13.5) | 333 (15.5) | 212 (21.3) |
| Race/Ethnicity (n (%)) |  |  |  |  |  |
| White/Aus | 452 (83.5) | 2120 (85.9) | 3381 (85.4) | 1798 (83.5) | 771 (77.5) |
| White/US | 45 (8.3) | 197 (8.0) | 287 (7.2) | 110 (5.1) | 58 (5.8) |
| Black | 18 (3.3) | 57 (2.3) | 173 (4.4) | 153 (7.1) | 117 (11.8) |
| Hispanic | 9 (1.7) | 49 (2.0) | 78 (2.0) | 74 (3.4) | 43 (4.3) |
| Other | 17 (3.1) | 46 (1.9) | 40 (1.0) | 18 (0.8) | 6 (0.6) |
| Weight in kg (mean (SD))^1^ | 50.7 (4.9) | 59.7 (5.3) | 69.6 (6.4) | 81.3 (7.2) | 96.9 (11.3) |
| Height in m (mean (SD))^2^ | 1.6 (0.1) | 1.6 (0.1) | 1.6 (0.1) | 1.6 (0.1) | 1.6 (0.1) |
| BMI in kg/m² (mean (SD))^3^ | 19.7 (1.1) | 23.3 (1.1) | 27.4 (1.4) | 32.1 (1.4) | 38.6 (3.4) |
| Waist Circum. in cm (mean (SD))^4^ | 73.5 (6.4) | 82.9 (7.3) | 92.2 (7.7) | 101.9 (8.1) | 113.5 (10.7) |
| Randomized to Aspirin (n (%)) | 260 (48.1) | 1251 (50.7) | 1959 (49.5) | 1047 (48.6) | 517 (52.0) |
| Baseline statin use (n (%)) | 97 (17.9) | 654 (26.5) | 1395 (35.2) | 847 (39.3) | 414 (41.6) |
| Current Smoker (n (%)) | 33 (6.1) | 108 (4.4) | 107 (2.7) | 54 (2.5) | 23 (2.3) |
| Current alcohol consumption (n (%)) | 400 (73.9) | 1890 (76.5) | 2956 (74.7) | 1427 (66.3) | 635 (63.8) |
| Diabetes (n (%)) | 10 (1.8) | 107 (4.3) | 292 (7.4) | 291 (13.5) | 198 (19.9) |
| Systolic BP (mean (SD)) | 135.2 (18.3) | 137.1 (17.6) | 138.2 (16.2) | 138 (16.5) | 137.5 (16.7) |
| Diastolic BP (mean (SD)) | 73.6 (10.7) | 75.3 (10.1) | 76.9 (10.1) | 77.9 (10.1) | 77.8 (10.1) |
| Education Level (n (%)) |  |  |  |  |  |
| <12 years | 222 (41.0) | 1065 (43.1) | 1846 (46.6) | 1016 (47.2) | 492 (49.4) |
| ≥12 years | 319 (59.0) | 1404 (56.9) | 2113 (53.4) | 1137 (52.8) | 503 (50.6) |
| Living Situation (n (%)) |  |  |  |  |  |
| At home alone | 249 (46.0) | 1054 (42.7) | 1585 (40.0) | 927 (43.1) | 443 (44.5) |
| With others | 292 (54.0) | 1415 (57.3) | 2374 (60.0) | 1226 (56.9) | 552 (55.5) |
| Longest amount of time walking outside home without any rest (last 2 weeks) | | | | | |
| < 10 minutes | 12 (2.2) | 29 (1.2) | 110 (2.8) | 68 (3.2) | 85 (8.5) |
| 10-15 minutes | 33 (6.1) | 188 (7.6) | 374 (9.4) | 285 (13.2) | 207 (20.8) |
| 16-30 minutes | 106 (19.6) | 560 (22.7) | 908 (22.9) | 615 (28.6) | 303 (30.5) |
| More than 30 minutes | 390 (72.1) | 1692 (68.5) | 2567 (64.8) | 1185 (55) | 400 (40.2) |
| Haemoglobin, g/dL (mean (SD))^5^ | 13.6 (1.1) | 13.6 (1.0) | 13.6 (1.0) | 13.7 (1.0) | 13.6 (1.0) |
| HDL-c, mmol/L (mean (SD))^6^ | 2.0 (0.5) | 1.9 (0.5) | 1.7 (0.4) | 1.6 (0.4) | 1.5 (0.4) |
| Non-HDL-c, mmol/L (mean (SD))^7^ | 3.0 (0.8) | 3.1 (0.9) | 3.1 (0.9) | 3.0 (0.9) | 3.0 (0.9) |
| Serum creatinine, mg/dL (mean (SD))^8^ | 0.8 (0.2) | 0.8 (0.2) | 0.8 (0.2) | 0.8 (0.2) | 0.8 (0.2) |
| Family history of MI (%) | 237 (43.8) | 1092 (44.2) | 1895 (47.9) | 1000 (46.4) | 462 (46.4) |
| Previous cancer diagnosis^9^ | 105 (19.4) | 435 (17.6) | 710 (17.9) | 332 (15.4) | 158 (15.9) |
| IRSAD score (mean (SD))^10^ | 1013.6 (70.9) | 1010 (69.1) | 1003.6 (68.1) | 997.5 (68.4) | 988 (68.5) |
| Intake of antihypertensive agents (%) | 203 (37.5) | 1070 (43.3) | 2119 (53.5) | 1402 (65.1) | 757 (76.1) |

Missing values for variables were: ¹n = 0, ²n = 0, ³n = 0, ⁴n = 122, ⁵n = 0, ⁶n = 231, ⁷n = 248, ⁸n = 233, ⁹n = 9, ¹⁰n = 1474.

Abbreviations: SD, standard deviation; BMI, body mass index; BP, blood pressure, MI = myocardial infarction, IRSAD = the Index of Relative Socio-economic Advantage and Disadvantage, HDL-c = high-density lipoprotein cholesterol.

Diabetes defined as a self-report, fasting glucose ≥ 126 mg/dL, or receiving pharmacologic treatment for diabetes (regardless of fasting glucose level).

**Appendices Table 2.** Baseline characteristics of the ASPREE participants by BMI category in men (n = 8,032)

|  | **BMI < 21.0** | **BMI 21.0-24.9** | **BMI 25.0-29.9** | **BMI 30.0-34.9** | **BMI 35.0+** |
| --- | --- | --- | --- | --- | --- |
|  | n=153 | n=1646 | n=4183 | n=1633 | n=417 |
| Age (mean (SD)) | 75.5 (5.1) | 75.6 (4.8) | 75.1 (4.4) | 74.2 (4.0) | 73.6 (3.5) |
| Country (n (%)) |  |  |  |  |  |
| Australia | 129 (84.3) | 1485 (90.2) | 3823 (91.4) | 1479 (90.6) | 352 (84.4) |
| United States | 24 (15.7) | 161 (9.8) | 360 (8.6) | 154 (9.4) | 65 (15.6) |
| Race/Ethnicity (n (%)) |  |  |  |  |  |
| White/Aus | 120 (78.4) | 1426 (86.6) | 3744 (89.5) | 1449 (88.7) | 347 (83.2) |
| White/US | 5 (3.3) | 81 (4.9) | 170 (4.1) | 56 (3.4) | 24 (5.8) |
| Black | 18 (11.8) | 57 (3.5) | 120 (2.9) | 60 (3.7) | 28 (6.7) |
| Hispanic | 1 (0.7) | 22 (1.3) | 100 (2.4) | 55 (3.4) | 13 (3.1) |
| Other | 9 (5.9) | 60 (3.6) | 49 (1.2) | 13 (0.8) | 5 (1.2) |
| Weight in kg (mean (SD))^1^ | 59.6 (6.1) | 71 (6.3) | 82.3 (7.4) | 95.4 (8.4) | 112.3 (11.6) |
| Height in m (mean (SD))^2^ | 1.7 (0.1) | 1.7 (0.1) | 1.7 (0.1) | 1.7 (0.1) | 1.7 (0.1) |
| BMI in kg/m² (mean (SD))^3^ | 19.8 (1.1) | 23.6 (1.0) | 27.4 (1.4) | 31.9 (1.3) | 37.7 (2.7) |
| Waist Circum. in cm (mean (SD))^4^ | 82.8 (6.8) | 91.7 (5.9) | 100.7 (6.4) | 111.1 (7.0) | 124.2 (9.4) |
| Randomized to Aspirin (n (%)) | 78 (51.0) | 830 (50.4) | 2092 (50.0) | 798 (48.9) | 215 (51.6) |
| Baseline statin use (n (%)) | 23 (15.0) | 336 (20.4) | 1153 (27.6) | 552 (33.8) | 157 (37.6) |
| Current Smoker (n (%)) | 27 (17.6) | 100 (6.1) | 162 (3.9) | 62 (3.8) | 14 (3.4) |
| Current alcohol consumption (n (%)) | 120 (78.4) | 1373 (83.4) | 3523 (84.2) | 1370 (83.9) | 328 (78.7) |
| Diabetes (n (%)) | 6 (3.9) | 129 (7.8) | 442 (10.6) | 288 (17.6) | 125 (30.0) |
| Systolic BP (mean (SD)) | 136.2 (18.7) | 140.1 (16.7) | 141.3 (15.6) | 142 (15.6) | 141.9 (14.2) |
| Diastolic BP (mean (SD)) | 75.1 (10.4) | 76.5 (9.5) | 78.2 (9.5) | 79.4 (9.6) | 78.8 (9.2) |
| Education Level (n (%)) |  |  |  |  |  |
| <12 years | 62 (40.5) | 645 (39.2) | 1809 (43.2) | 782 (47.9) | 204 (48.9) |
| ≥12 years | 91 (59.5) | 1001 (60.8) | 2374 (56.8) | 851 (52.1) | 213 (51.1) |
| Living Situation (n (%)) |  |  |  |  |  |
| At home alone | 54 (35.3) | 376 (22.8) | 808 (19.3) | 330 (20.2) | 81 (19.4) |
| With others | 99 (64.7) | 1270 (77.2) | 3375 (80.7) | 1303 (79.8) | 336 (80.6) |
| Longest amount of time walking outside home without any rest (last 2 weeks) | | | | | |
| < 10 minutes | 4 (2.6) | 28 (1.7) | 108 (2.6) | 59 (3.6) | 30 (7.2) |
| 10-15 minutes | 18 (11.8) | 127 (7.7) | 314 (7.5) | 172 (10.5) | 78 (18.7) |
| 16-30 minutes | 34 (22.2) | 306 (18.6) | 836 (20) | 351 (21.5) | 114 (27.3) |
| More than 30 minutes | 97 (63.4) | 1185 (72) | 2925 (69.9) | 1051 (64.4) | 195 (46.8) |
| Haemoglobin, g/dL (mean (SD))^5^ | 14.3 (1.0) | 14.7 (1.1) | 14.9 (1.1) | 15 (1.1) | 14.9 (1.3) |
| HDL-c, mmol/L (mean (SD))^6^ | 1.7 (0.5) | 1.6 (0.4) | 1.4 (0.4) | 1.3 (0.3) | 1.2 (0.3) |
| Non-HDL-c, mmol/L (mean (SD))^7^ | 2.9 (0.8) | 3.0 (0.8) | 3.1 (0.8) | 2.9 (0.9) | 2.8 (0.8) |
| Serum creatinine, mg/dL (mean (SD))^8^ | 0.9 (0.2) | 1.0 (0.2) | 1.0 (0.2) | 1.0 (0.2) | 1.1 (0.2) |
| Family history of MI (%) | 57 (37.3) | 597 (36.3) | 1634 (39.1) | 618 (37.8) | 175 (42) |
| Previous cancer diagnosis^9^ | 18 (11.8) | 350 (21.3) | 899 (21.5) | 377 (23.1) | 89 (21.3) |
| IRSAD score (mean (SD))^10^ | 1000.6 (68.5) | 1009.5 (69.5) | 1004.4 (68.9) | 1001.6 (70.4) | 986.4 (67.2) |
| Intake of antihypertensive agents (%) | 42 (27.5) | 590 (35.8) | 1983 (47.4) | 986 (60.4) | 314 (75.3) |

Missing values for variables were: ¹n = 0, ²n = 0, ³n = 0, ⁴n = 50, ⁵n = 2, ⁶n = 201, ⁷n = 237, ⁸n = 211, ⁹n = 10, ¹⁰n = 786. Abbreviations: SD, standard deviation; BMI, body mass index; BP, blood pressure, MI = myocardial infarction, IRSAD = the Index of Relative Socio-economic Advantage and Disadvantage, HDL-c = high-density lipoprotein cholesterol.

Diabetes defined as a self-report, fasting glucose ≥ 126 mg/dL, or receiving pharmacologic treatment for diabetes (regardless of fasting glucose level).

**Appendices Table 3.** Baseline characteristics of the ASPREE participants by waist circumference quintile in women (n = 10,033)

|  | **Q 1** | **Q 2** | **Q 3** | **Q 4** | **Q 5** |
| --- | --- | --- | --- | --- | --- |
|  | n=2087 | n=2027 | n=2127 | n=1985 | n=1807 |
| Age (mean (SD)) | 75.5 (4.6) | 75.4 (4.6) | 75.3 (4.6) | 75.0 (4.5) | 74.8 (4.4) |
| Country (n (%)) |  |  |  |  |  |
| Australia | 1812 (86.8) | 1775 (87.6) | 1858 (87.4) | 1685 (84.9) | 1476 (81.7) |
| United States | 275 (13.2) | 252 (12.4) | 269 (21.6) | 300 (15.1) | 331 (18.3) |
| Race/Ethnicity (n (%)) |  |  |  |  |  |
| White/Aus | 1762 (84.4) | 1740 (85.8) | 1840 (86.5) | 1665 (83.9) | 1452 (80.4) |
| White/US | 179 (8.6) | 144 (7.1) | 138 (6.5) | 120 (6) | 112 (6.2) |
| Black | 62 (3) | 75 (3.7) | 85 (4) | 121 (6.1) | 165 (9.1) |
| Hispanic | 35 (1.7) | 36 (1.8) | 49 (2.3) | 61 (3.1) | 64 (3.5) |
| Other | 49 (2.3) | 32 (1.6) | 15 (0.7) | 18 (0.9) | 14 (0.8) |
| Weight in kg (mean (SD))^1^ | 57.5 (7.1) | 64.9 (6.9) | 70.3 (7.5) | 77.2 (8.8) | 89.4 (12.5) |
| Height in m (mean (SD))^2^ | 1.6 (0.1) | 1.6 (0.1) | 1.6 (0.1) | 1.6 (0.1) | 1.6 (0.1) |
| BMI in kg/m² (mean (SD))^3^ | 22.8 (2.6) | 25.7 (2.5) | 27.6 (2.9) | 30.3 (3.2) | 34.9 (4.8) |
| Waist Circum. in cm (mean (SD))^4^ | 76.2 (4.9) | 86.1 (2.0) | 93.0 (2.0) | 100.2 (2.3) | 112.8 (7.7) |
| Randomized to Aspirin (n (%)) | 1066 (51.1) | 992 (48.9) | 1055 (49.6) | 992 (50.0) | 886 (49.0) |
| Baseline statin use (n (%)) | 514 (24.6) | 622 (30.7) | 744 (35) | 771 (38.8) | 744 (41.2) |
| Current Smoker (n (%)) | 86 (4.1) | 67 (3.3) | 63 (3.0) | 58 (2.9) | 45 (2.5) |
| Current alcohol consumption (n (%)) | 1538 (73.7) | 1530 (75.5) | 1586 (74.6) | 1404 (70.7) | 1193 (66.0) |
| Diabetes (n (%)) | 73 (3.5) | 84 (4.1) | 175 (8.2) | 234 (11.8) | 324 (17.9) |
| Systolic BP (mean (SD)) | 136.5 (18.0) | 137.5 (16.5) | 138.7 (16.6) | 137.5 (16.4) | 138 (16.3) |
| Diastolic BP (mean (SD)) | 74.8 (10.3) | 76.3 (9.9) | 77.4 (10.1) | 77.2 (10.1) | 77.6 (10.2) |
| Education Level (n (%)) |  |  |  |  |  |
| <12 years | 897 (43.0) | 927 (45.7) | 958 (45.0) | 958 (48.3) | 866 (47.9) |
| ≥12 years | 1190 (57.0) | 1100 (54.3) | 1169 (55.0) | 1027 (51.7) | 941 (52.1) |
| Living Situation (n (%)) |  |  |  |  |  |
| At home alone | 859 (41.2) | 860 (42.4) | 849 (39.9) | 856 (43.1) | 800 (44.3) |
| With others | 1228 (58.8) | 1167 (57.6) | 1278 (60.1) | 1129 (56.9) | 1007 (55.7) |
| Longest amount of time walking outside home without any rest (last 2 weeks) | | | | | |
| < 10 minutes | 32 (1.5) | 37 (1.8) | 50 (2.4) | 71 (3.6) | 107 (5.9) |
| 10-15 minutes | 142 (6.8) | 167 (8.2) | 207 (9.7) | 248 (12.5) | 314 (17.4) |
| 16-30 minutes | 435 (20.8) | 428 (21.1) | 519 (24.4) | 542 (27.3) | 542 (30.0) |
| More than 30 minutes | 1478 (70.8) | 1395 (68.8) | 1351 (63.5) | 1124 (56.6) | 844 (46.7) |
| Haemoglobin, g/dL (mean (SD))^5^ | 13.6 (1.0) | 13.6 (1.0) | 13.6 (1.0) | 13.6 (1.0) | 13.7 (1.1) |
| HDL-c, mmol/L (mean (SD))^6^ | 2.0 (0.5) | 1.8 (0.4) | 1.7 (0.4) | 1.6 (0.4) | 1.5 (0.4) |
| Non-HDL-c, mmol/L (mean (SD))^7^ | 3.1 (0.8) | 3.1 (0.9) | 3.1 (0.9) | 3.1 (0.9) | 3.0 (0.9) |
| Serum creatinine, mg/dL (mean (SD))^8^ | 0.8 (0.2) | 0.8 (0.2) | 0.8 (0.2) | 0.8 (0.2) | 0.8 (0.2) |
| Family history of MI (%) | 923 (44.2) | 958 (47.3) | 998 (46.9) | 920 (46.3) | 846 (46.8) |
| Previous cancer diagnosis^9^ | 368 (17.6) | 378 (18.6) | 364 (17.1) | 299 (15.1) | 322 (17.8) |
| IRSAD score (mean (SD))^10^ | 1006.8 (68.9) | 1004.6 (68.8) | 1005.6 (68.9) | 1000.2 (68) | 995.1 (68.6) |
| Intake of antihypertensive agents (%) | 849 (40.7) | 1002 (49.4) | 1135 (53.4) | 1249 (62.9) | 1273 (70.4) |

Missing values for variables were: ¹n = 23, ²n = 17, ³n = 38, ⁴n = 0, ⁵n = 0, ⁶n = 230, ⁷n = 247, ⁸n = 229, ⁹n = 9, ¹⁰n = 1452.
Abbreviations: SD, standard deviation; BMI, body mass index; BP, blood pressure; MI = myocardial infarction, IRSAD = the Index of Relative Socio-economic Advantage and Disadvantage, HDL-c = high-density lipoprotein cholesterol.

Diabetes defined as a self-report, fasting glucose ≥ 126 mg/dL, or receiving pharmacologic treatment for diabetes (regardless of fasting glucose level).

**Appendices Table 4.** Baseline characteristics of the ASPREE participants by waist circumference quintile in men (n = 8,004)

|  | **Quintile 1** | **Quintile 2** | **Quintile 3** | **Quintile 4** | **Quintile 5** |
| --- | --- | --- | --- | --- | --- |
|  | n=1668 | n=1828 | n=1594 | n=1400 | n=1514 |
| Age (mean (SD)) | 75.1 (4.6) | 75.2 (4.5) | 75.1 (4.6) | 74.8 (4.3) | 74.3 (4.0) |
| Country (n (%)) |  |  |  |  |  |
| Australia | 1493 (89.5) | 1680 (91.9) | 1441 (90.4) | 1277 (91.2) | 1364 (90.1) |
| United States | 175 (10.5) | 148 (8.1) | 153 (9.6) | 123 (8.8) | 150 (9.9) |
| Race/Ethnicity (n (%)) |  |  |  |  |  |
| White/Aus | 1422 (85.3) | 1645 (90.0) | 1404 (88.1) | 1253 (89.5) | 1349 (89.1) |
| White/US | 71 (4.3) | 74 (4.0) | 73 (4.6) | 46 (3.3) | 63 (4.2) |
| Black | 80 (4.8) | 42 (2.3) | 52 (3.3) | 43 (3.1) | 61 (4.0) |
| Hispanic | 31 (1.9) | 36 (2.0) | 46 (2.9) | 43 (3.1) | 33 (2.2) |
| Other | 64 (3.8) | 31 (1.7) | 19 (1.2) | 15 (1.1) | 8 (0.5) |
| Weight in kg (mean (SD))^1^ | 70.3 (7.7) | 77.9 (6.5) | 83.4 (6.9) | 88.9 (7.6) | 101.2 (11.4) |
| Height in m (mean (SD))^2^ | 1.7 (0.1) | 1.7 (0.1) | 1.7 (0.1) | 1.7 (0.1) | 1.7 (0.1) |
| BMI in kg/m² (mean (SD))^3^ | 24.0 (2.3) | 26.2 (2) | 27.8 (2) | 29.4 (2.2) | 33.1 (3.5) |
| Waist Circum. in cm (mean (SD))^4^ | 88.1 (4.6) | 96.7 (1.7) | 102 (1.4) | 107.3 (1.7) | 118.1 (7.0) |
| Randomized to Aspirin (n (%)) | 840 (50.4) | 926 (50.7) | 774 (48.6) | 696 (49.7) | 766 (50.6) |
| Baseline statin use (n (%)) | 347 (20.8) | 464 (25.4) | 420 (26.3) | 439 (31.4) | 543 (35.9) |
| Current Smoker (n (%)) | 115 (6.9) | 69 (3.8) | 64 (4.0) | 54 (3.9) | 59 (3.9) |
| Current alcohol consumption (n (%)) | 1359 (81.5) | 1554 (85.0) | 1347 (84.5) | 1177 (84.1) | 1255 (82.9) |
| Diabetes (n (%)) | 110 (6.6) | 161 (8.8) | 185 (11.6) | 207 (14.8) | 322 (21.3) |
| Systolic BP (mean (SD)) | 139.7 (16.6) | 141.7 (16) | 141.8 (15.7) | 141.7 (15.5) | 140.9 (15.1) |
| Diastolic BP (mean (SD)) | 76.8 (9.6) | 78 (9.5) | 78.2 (9.6) | 78.9 (9.6) | 78.8 (9.5) |
| Education Level (n (%)) |  |  |  |  |  |
| <12 years | 661 (39.6) | 775 (42.4) | 688 (43.2) | 649 (46.4) | 727 (48.0) |
| ≥12 years | 1007 (60.4) | 1053 (57.6) | 906 (56.8) | 751 (53.6) | 787 (52.0) |
| Living Situation (n (%)) |  |  |  |  |  |
| At home alone | 375 (22.5) | 359 (19.6) | 314 (19.7) | 280 (20.0) | 317 (20.9) |
| With others | 1293 (77.5) | 1469 (80.4) | 1280 (80.3) | 1120 (80.0) | 1197 (79.1) |
| Longest amount of time walking outside home without any rest (last 2 weeks) | | | | | |
| < 10 minutes | 24 (1.4) | 27 (1.5) | 44 (2.8) | 55 (3.9) | 76 (5.0) |
| 10-15 minutes | 105 (6.3) | 134 (7.3) | 124 (7.8) | 144 (10.3) | 197 (13.0) |
| 16-30 minutes | 294 (17.6) | 351 (19.2) | 333 (20.9) | 275 (19.6) | 386 (25.5) |
| More than 30 minutes | 1245 (74.6) | 1316 (72.0) | 1093 (68.6) | 926 (66.1) | 855 (56.5) |
| Haemoglobin, g/dL (mean (SD))^5^ | 14.7 (1.1) | 14.9 (1.1) | 15.0 (1.1) | 15.0 (1.1) | 14.9 (1.2) |
| HDL-c, mmol/L (mean (SD))^6^ | 1.6 (0.4) | 1.4 (0.4) | 1.4 (0.4) | 1.3 (0.4) | 1.3 (0.3) |
| Non-HDL-c, mmol/L (mean (SD))^7^ | 3.0 (0.9) | 3.1 (0.8) | 3.0 (0.8) | 3.0 (0.8) | 2.9 (0.9) |
| Serum creatinine, mg/dL (mean (SD))^8^ | 1.0 (0.2) | 1.0 (0.2) | 1.0 (0.2) | 1.0 (0.2) | 1.0 (0.2) |
| Family history of MI (%) | 615 (36.9) | 701 (38.3) | 615 (38.6) | 550 (39.3) | 594 (39.2) |
| Previous cancer diagnosis^9^ | 303 (18.2) | 399 (21.8) | 351 (22.0) | 317 (22.6) | 362 (23.9) |
| IRSAD score (mean (SD))^10^ | 1006.7 (69.9) | 1005.5 (68.5) | 1005.1 (69.5) | 1003.9 (68.7) | 997.1 (69.8) |
| Intake of antihypertensive agents (%) | 569 (34.1) | 817 (44.7) | 774 (48.6) | 770 (55.0) | 983 (64.9) |

Missing values for variables were: ¹n = 12, ²n = 11, ³n = 22, ⁴n = 0, ⁵n = 2, ⁶n = 201, ⁷n = 237, ⁸n = 211, ⁹n = 10, ¹⁰n = 771.
Abbreviations: SD, standard deviation; BMI, body mass index; BP, blood pressure; MI = myocardial infarction, IRSAD = the Index of Relative Socio-economic Advantage and Disadvantage, HDL-c = high-density lipoprotein cholesterol.

Diabetes defined as a self-report, fasting glucose ≥ 126 mg/dL, or receiving pharmacologic treatment for diabetes (regardless of fasting glucose level).

**Appendices Table 5.** **Hazard ratio (95% CI) of all-cause mortality and cause specific mortality according to WHO body mass index categories in men and women (n=18,149)**

|  | **All-cause mortality** | | | **Cancer death** | | | **Cardiovascular death** | | | **Other death** | | |
| --- | --- | --- | --- | --- | --- | --- | --- | --- | --- | --- | --- | --- |
| **BMI (kg/m^2^)** | No. of deaths/No. of pts | Incidence rate per 1000py | HR^1^  (95% CI) | No. of deaths/No. of pts | Incidence rate per 1000py | HR^1^  (95% CI) | No. of deaths/No. of pts | Incidence rate per 1000py | HR^1^  (95% CI) | No. of deaths/No. of pts | Incidence rate per 1000py | HR^1^  (95% CI) |
| **Men** |  |  |  |  |  |  |  |  |  |  |  |  |
| **<18.5** | 8/19 | 77.41 | 3.74 (1.85, 7.59) | 1/19 | 9.68 | 1.09 (0.15, 7.84) | 3/19 | 29.03 | 7.02 (2.17, 22.68) | 4/19 | 38.71 | 5.44 (1.99, 14.90) |
| **18.5-24.9** | 265/1780 | 22.79 | Ref | 103/1780 | 8.86 | Ref | 56/1780 | 4.82 | Ref | 101/1780 | 8.69 | Ref |
| **25 – 29.9** | 458/4183 | 16.50 | 0.81 (0.70, 0.95) | 228/4183 | 8.21 | 1.01 (0.80, 1.28) | 84/4183 | 3.03 | 0.70 (0.50, 0.99) | 138/4183 | 4.97 | 0.67 (0.51, 0.87) |
| **≥30** | 231/2050 | 17.20 | 0.91 (0.76, 1.09) | 118/2050 | 8.79 | 1.14 (0.87, 1.50) | 53/2050 | 3.95 | 1.00 (0.68, 1.48) | 60/2050 | 4.47 | 0.67 (0.48, 0.93) |
| **Women** |  |  |  |  |  |  |  |  |  |  |  |  |
| **<18.5** | 18/78 | 35.42 | 2.47 (1.53, 3.99) | 6/78 | 11.81 | 2.16 (0.94, 4.92) | 2/78 | 3.94 | 1.43 (0.34, 5.93) | 10/78 | 19.68 | 3.47 (1.80, 6.67) |
| **18.5-24.9** | 255/2932 | 12.71 | Ref | 100/2932 | 4.98 | Ref | 44/2932 | 2.19 | Ref | 104/2932 | 5.18 | Ref |
| **25 – 29.9** | 287/3959 | 10.70 | 0.90 (0.76, 1.07) | 147/3959 | 5.48 | 1.16 (0.90, 1.49) | 54/3959 | 2.01 | 0.99 (0.66, 1.48) | 84/3959 | 3.13 | 0.65 (0.49, 0.87) |
| **≥30** | 234/3148 | 11.12 | 0.99 (0.82, 1.19) | 114/3148 | 5.42 | 1.17 (0.88, 1.55) | 50/3148 | 2.38 | 1.34 (0.88, 2.05) | 66/3148 | 3.14 | 0.69 (0.50, 0.96) |

^1^HRs adjusted for age, smoking status, aspirin treatment arm, diabetes status (yes, no), level of education (<12 years, ≥12 years), living status (at home alone, with others), alcohol consumption (current alcohol consumption, former/never), and longest amount of time walking outside home without any rest.

Abbreviations: BMI, body mass index; py, person years; HR, hazard ratio; CI, confidence interval; Ref, reference.

**Appendices Table 6. Hazard ratio (95% CI) of all cause and cause-specific mortality according to WHO waist circumference cut-offs in men and women (n = 18,307)**

|  | **All-cause mortality** | | | **Cancer death** | | | **Cardiovascular death** | | | **Other death** | | |
| --- | --- | --- | --- | --- | --- | --- | --- | --- | --- | --- | --- | --- |
| **Waist circumference (cm)** | No. of deaths/No. of pts | Incidence rate per 1000py | HR^1^  (95% CI) | No. of deaths/No. of pts | Incidence rate per 1000py | HR^1^  (95% CI) | No. of deaths/No. of pts | Incidence rate per 1000py | HR^1^  (95% CI) | No. of deaths/No. of pts | Incidence rate per 1000py | HR^1^  (95% CI) |
| **Men** |  |  |  |  |  |  |  |  |  |  |  |  |
| **< 102** | 488/4137 | 17.80 | Ref | 221/4137 | 8.06 | Ref | 89/4137 | 3.25 | Ref | 170/4137 | 6.20 | Ref |
| **≥ 102** | 466/3867 | 18.40 | 1.07 (0.94, 1.22) | 227/3867 | 8.96 | 1.13 (0.93, 1.36) | 104/3867 | 4.11 | 1.31 (0.98, 1.75) | 131/3867 | 5.17 | 0.89 (0.70, 1.12) |
| **Women** |  |  |  |  |  |  |  |  |  |  |  |  |
| **< 88** | 270/3503 | 11.29 | Ref | 117/3503 | 4.89 | Ref | 41/3503 | 1.71 | Ref | 103/3503 | 4.31 | Ref |
| **≥ 88** | 510/6530 | 11.62 | 1.05 (0.90, 1.22) | 245/6530 | 5.58 | 1.15 (0.92, 1.44) | 105/6530 | 2.39 | 1.45 (1.00, 2.09) | 155/6530 | 3.53 | 0.84 (0.65, 1.09) |

^1^HRs adjusted for age, smoking status, aspirin treatment arm, diabetes status (yes, no), level of education (<12 years, ≥12 years), living status (at home alone, with others), alcohol consumption (current alcohol consumption, former/never), and longest amount of time walking outside home without any rest.

Abbreviations: py, person years; HR, hazard ratio; CI, confidence interval; Ref, reference.
